# Prognostic Significance of AI-Enhanced ECG for Emergency Department Patients

**DOI:** 10.3390/diagnostics15151874

**Published:** 2025-07-25

**Authors:** Yu-Te Su, Sy-Jou Chen, Chin Lin, Chin-Sheng Lin, Hsiao-Feng Hu

**Affiliations:** 1 Department of Emergency Medicine, Tri-Service General Hospital, National Defense Medical University, Taipei 11490, Taiwan; m42k6nj@gmail.com (Y.-T.S.); syjou.chen@gmail.com (S.-J.C.); 2 Graduate Institute of Injury Prevention and Control, College of Public Health and Nutrition, Taipei Medical University, New Taipei City 235, Taiwan; 3 School of Public Health, National Defense Medical University, Taipei 11490, Taiwan; xup6fup0629@gmail.com; 4 Department of Artificial Intelligence and Internet of Things, Tri-Service General Hospital, National Defense Medical University, Taipei 11490, Taiwan; 5 Medical Technology Education Center, School of Medicine, National Defense Medical University, Taipei 11490, Taiwan; littlelincs@gmail.com; 6 Graduate Institute of Aerospace and Undersea Medicine, National Defense Medical University, Taipei 11490, Taiwan; 7 Division of Cardiology, Department of Internal Medicine, Tri-Service General Hospital, National Defense Medical University, Taipei 11490, Taiwan; 8 Tri-Service General Hospital Penghu Branch, National Defense Medical University, Penghu 880026, Taiwan; 9 Graduate Institute of Applied Science and Technology, National Taiwan University of Science and Technology, Taipei 10607, Taiwan

**Keywords:** artificial intelligence, electrocardiogram, emergency department, mortality

## Abstract

**Background/Objectives**: Artificial intelligence (AI)-enabled electrocardiogram (ECG) analysis may assist in objective and reproducible risk stratification. However, the prognostic utility of serial ECGs, particularly the follow-up ECG prior to discharge, has not been extensively studied. This study aimed to evaluate whether dynamic changes in AI-predicted ECG risk scores could enhance prediction of post-discharge outcomes. **Methods**: This retrospective cohort study included 11,508 ED visits from a single medical center where patients underwent two ECGs and were directly discharged. We stratified the mortality risk of patients as low risk, medium risk, and high risk based on the first and follow-up ECG prior to discharge using AI-enabled ECG models. The Area Under the Curve (AUC) was calculated for the predictive performance of the two ECGs. Kaplan–Meier (KM) curves were used for 90-day mortality analysis, and the Cox proportional hazards model was utilized to compare the risk of death across categories. **Results**: The AI-enabled ECG risk prediction model, based on the initial and follow-up ECGs prior to discharge, indicated risk transitions among different groups. The AUC for mortality risk was 78.6% for the first ECG and 83.3% for the follow-up ECG. KM curves revealed a significant increase in 90-day mortality for patients transitioning from low to medium/high risk upon discharge (Hazard Ratio: 6.01; Confidence Interval: 1.70–21.27). **Conclusions**: AI-enabled ECGs obtained prior to discharge provide superior mortality risk stratification for ED patients compared to initial ECGs. Patients classified as medium- or high-risk at discharge require careful consideration, whereas those at low risk can generally be discharged safely. Although AI-ECG alone does not replace comprehensive risk assessment, it offers a practical tool to support clinical judgment, particularly in the dynamic ED environment, by aiding safer discharge decisions.

## 1. Introduction

Efficient patient disposition is an important challenge in emergency departments (EDs), where clinicians must balance the need for prompt care with the imperative to accurately assess risk. Despite thorough assessment and treatment, patients discharged from the ED may experience adverse outcomes that were not anticipated at the time of discharge. A previous study found that the overall 72 h revisit rate to the ED was 5.47% [1]. Furthermore, missed and delayed diagnoses in the ED can lead to serious adverse outcomes, including death, with stroke, myocardial infarction, and aortic aneurysm/dissection among the most commonly overlooked [2]. These statistics highlight the challenge of accurately identifying patients at elevated risk during the disposition process and underscore the need for more effective risk stratification tools.

The discharge process from the ED involves a complex interplay among patient factors, clinician judgment, and system constraints [3]. Traditional diagnostic tools and risk scores, while valuable, ultimately rely on clinician interpretation and can sometimes fail to capture a patient’s true clinical risk, potentially resulting in missed diagnoses or inappropriate disposition [4]. This limitation is particularly evident in the interpretation of ECGs, which have become fundamental to risk assessment in the ED, especially for patients with cardiovascular history or presenting with symptoms such as chest pain or dyspnea. In addition to conventional ECG interpretation, heart rate variability (HRV) measures such as the standard deviation of normal-to-normal intervals (SDNN) and very low frequency (VLF) have been established as predictors [5]. However, HRV captures limited aspects of cardiac dynamics and requires preprocessing, whereas deep learning models can directly analyze raw waveforms to extract richer prognostic information. This highlights the need for more objective and reliable methods to enhance ECG analysis and improve patient risk stratification.

Artificial intelligence (AI) has emerged as a promising solution to these challenges by offering capabilities that extend beyond traditional clinical assessment. Advanced AI algorithms can detect subtle patterns and abnormalities in ECG data that might escape human observation, enabling prediction of various cardiovascular conditions including heart failure, atrial fibrillation, and overall mortality risk [6,7,8]. Recent studies have demonstrated the practical application of these capabilities, showing that AI-ECG algorithms can effectively predict left ventricular systolic dysfunction and stratify in-hospital mortality risk for intensive care patients [9,10]. Furthermore, an AI-enabled ECG alert system has been shown to significantly improve patient outcomes, with one study reporting a 31% reduction in the relative risk of 90-day all-cause mortality compared to standard care based on a single ECG analysis [11]. These findings suggest that AI-enhanced ECG interpretation could provide a more accurate, data-driven foundation for clinical decision-making in the ED.

In the context of high-acuity EDs, where rapid and accurate evaluation is essential, AI-enhanced ECG interpretation could be particularly valuable. Current ED practices often involve performing multiple ECGs and serial laboratory tests to evaluate higher-risk patients, especially those presenting with cardiopulmonary symptoms. However, no studies have investigated the comparative prognostic value of initial versus follow-up ECGs during an ED visit, nor how AI-enhanced interpretation might optimize this process. This study aims to address this gap by assessing the prognostic value of changes in AI-enabled ECG risk stratification for ED patients. We hypothesize that the predictive accuracy of follow-up ECGs will be superior to that of initial ECGs, potentially offering a more reliable basis for disposition decisions and improving patient outcomes.

## 2. Materials and Methods

### 2.1. Study Design and Patients

This retrospective cohort study was conducted in the ED of Tri-Service General Hospital (TSGH) from July 2012 to December 2022. The study included patients who underwent at least two ECG examinations during their ED visit. The exclusion criteria were as follows: patients with a time interval exceeding 1 h from admission to the first ECG, as this interval may not accurately represent the initial condition; patients with a time interval of less than 1 h between the first and second ECGs, as this short interval may not allow for significant physiological changes to manifest; patients with a time interval exceeding 2 h from the second ECG to discharge, as this may not accurately reflect the condition at discharge; patients with a total ED stay exceeding 48 h, as this may involve more complex medical conditions and treatments; and patients who died during their ED visit. Patients who met inclusion criteria and were directly discharged alive formed the final study cohort. This study was approved by the Institutional Review Board of TSGH, Taipei, Taiwan (IRB No. C202105049), which waived the requirement for informed consent due to the retrospective nature of the research.

### 2.2. ECG Acquisition and Preprocessing

All electrocardiograms were acquired as standard 12-lead 10 s recordings using a digital ECG system with a sampling frequency of 500 Hz and an amplitude resolution of 5 microvolts per bit. The raw ECG waveforms underwent preprocessing that included bandpass filtering between 0.5 and 40 Hz to reduce baseline wander and high-frequency noise, followed by normalization to zero mean and unit variance across each lead. Each ECG was segmented into uniform 10 s matrices containing 12 leads, forming 5000 samples. In addition to waveform-level data, structured ECG features were extracted from machine-generated ECG interpretations using a standardized diagnostic classification system implemented in the Philips ECG platform, as described in our previous study [12]. These included 31 diagnostic pattern classes (e.g., atrial fibrillation, ventricular premature complexes, QT prolongation) and 8 continuous ECG measurements (e.g., heart rate, PR/QRS/QTc intervals, T/QRS axes). These features were used in secondary analyses to evaluate their potential association with AI-ECG risk predictions.

### 2.3. AI Model Architecture and Training

We developed a deep learning model based on an 82-layer convolutional neural network (CNN) architecture adapted from our previous studies [12], which originally demonstrated robust prognostic performance in large-scale ECG datasets. Briefly, the network incorporated multiple convolutional blocks with ReLU activations and batch normalization, followed by fully connected layers and a final sigmoid output neuron generating continuous probability scores. The dataset was partitioned by patient into development, tuning, and internal validation subsets to prevent cross-contamination. Model training was performed using the Adam optimizer (initial learning rate 0.001), a batch size of 32, L2 regularization, oversampling to address class imbalance, and early stopping based on validation loss. Model development and inference were implemented in Python 3.8 (TensorFlow and Keras). The model was trained on more than 450,000 ECGs with all-cause mortality as the primary outcome label, using survival data that included censored events. Its predictive performance was evaluated in an external validation dataset, achieving an Area Under the Curve (AUC) of 0.886 for 90-day mortality prediction. We applied gradient boosting models (XGBoost) using structured ECG features and patient demographics to estimate their relative importance in predicting AI-derived risk scores, as described in our earlier study [11]. A representative visual example of the AI model’s output and interpretability interface is presented in Figure S1 (Supplementary Materials).

### 2.4. AI-Enabled ECG Risk Stratification

For each ECG, the model generated a continuous probability score predicting 90-day all-cause mortality, which was transformed into percentile ranks relative to a hospital-wide reference population and categorized into three risk groups: low (<75th percentile), medium (75th–94th percentile), and high (≥95th percentile). For each patient, both the initial ECG at ED presentation and the follow-up ECG prior to discharge were analyzed. To assess temporal changes in risk classification, four transition groups were defined: low-to-low, medium/high-to-low, low-to-medium/high, and medium/high-to-medium/high. Patient survival status was determined using electronic medical records (EMRs). Although deaths occurring outside our institution may not have been captured, this limitation was considered minimal because only 0.16% of readmissions occurred at external hospitals during the reference period [13].

### 2.5. Statistical Analysis

The predictive performance of the AI-enabled ECG model was assessed by calculating the AUC. Cumulative survival probabilities within 90 days were estimated using Kaplan–Meier methods and compared across groups using the log-rank test. Associations between risk categories and all-cause mortality were assessed using Cox proportional hazards regression, and hazard ratios with 95% confidence intervals were reported. All statistical analyses were performed using R version 3.4.4, and a two-sided *p*-value less than 0.05 was considered statistically significant.

## 3. Results

### 3.1. Study Flowchart and AI-Enabled ECG Risk Categorization

**Figure 1:**
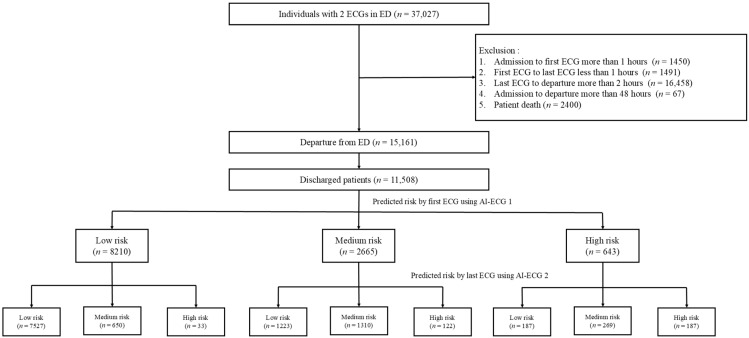
Case selection flowchart.

There were 37,027 patients who underwent at least two ECG examinations during the study period, and 21,866 patients were excluded. A total of 15,161 patients departed from the ED, with 11,508 patients discharged directly from the ED included in the final analysis. The AI-enabled ECG risk prediction model, based on the initial and follow-up ECGs prior to discharge, indicated transitions among different risk groups, as illustrated in Figure 1. Of note, numbers in the medium- and high-risk groups were decreased from 2665 and 643 to 2229 and 342 by subsequent AI-enabled ECG risk classification.

### 3.2. Baseline Characteristics of Patients

The demographic characteristics of these 11,508 patients are presented in Table 1. The mean age of the population was 63.6 ± 16.6 years, with males accounting for 53.6%. Level 3 triage accounted for the majority of cases (56.0%) in the ED. The mean AI-enabled ECG mortality risk score was 68.8 ± 24.1 for the first ECG, and 65.7 ± 24.1 for the subsequent ECG prior to discharge. The average duration from admission to discharge was 5.5 ± 4.4 h. Approximately 0.9% of patients experienced mortality during the 90-day follow-up period. The characteristics of the three risk categories predicted by AI-enabled ECG upon admission to the ED are detailed in Table 2. Significant differences were observed across the risk groups in age, sex distribution, vital signs, ECG-derived risk scores, triage levels, and clinical outcomes. Patients in the high-risk group were generally older (mean age: 69.98 years) and exhibited more severe triage classifications, with 10.3% assigned to level 1 acuity, compared to only 1.5% in the low-risk group. The second ECG mortality risk score, obtained prior to discharge, remained elevated in the high-risk cohort (mean: 87.4). The 90-day all-cause mortality rate increased proportionally with risk category: 0.3% in the low-risk group, 1.7% in the medium-risk group, and 3.9% in the high-risk group (*p* < 0.001). The characteristics of different risk transitions are presented in Appendix A Table A1, Table A2 and Table A3.

### 3.3. AI-Enabled ECG Risk Prediction

**Figure 2:**
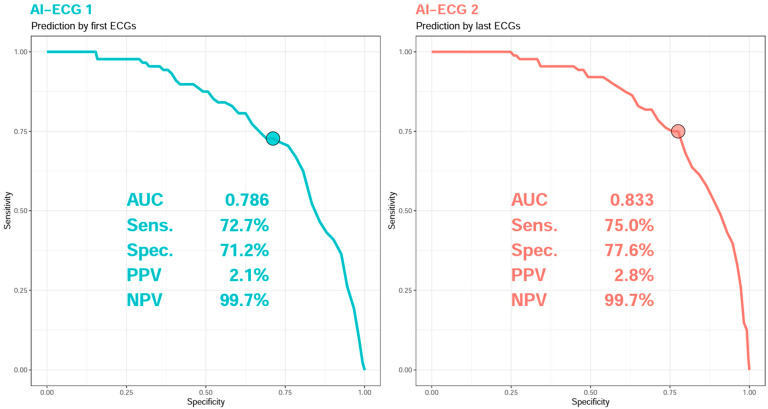
ROC curves of AI-enhanced ECG for predicting all-cause mortality within 90 days. The operating point was selected based on the maximum of Youden’s index in the tuning set and was presented using a circle mark.

Figure 2 illustrates the receiver operating characteristic (ROC) curves of the ECG prediction models of all-cause mortality within 90 days, which demonstrate that the AUC for the first and follow-up ECGs prior to discharge were 78.6% and 83.3%, respectively. The positive predictive values were 2.1% and 2.8%, respectively, while the negative predictive values were both 99.7%. The follow-up ECG indicated better predictive ability compared to the initial ECG.

### 3.4. Mortality Risk of AI-Enabled ECG Categorical Changes

**Figure 3:**
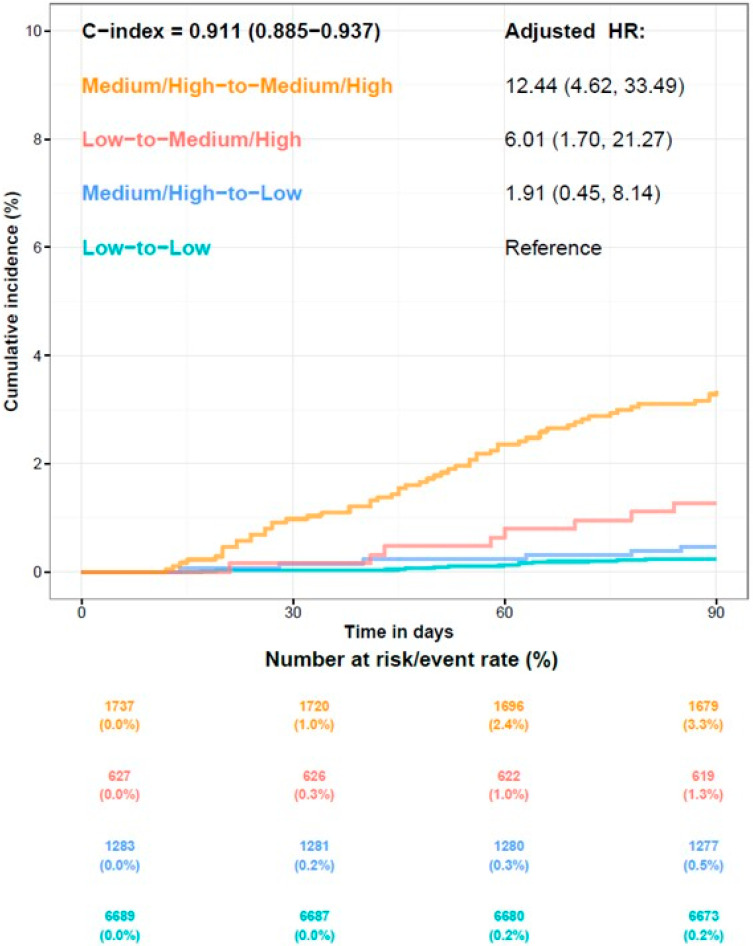
Kaplan–Meier curves for all-cause mortality in the 4 categories of patients within 90 days.

Cumulative death estimates for the four risk categories, based on risk scores generated by the AI model from the two ECGs, are shown as Kaplan–Meier curves in Figure 3. The incidences of 90-day mortality were 3.3% in the medium/high-to-medium/high risk group and 1.3% in the low-to-medium/high group, which were significantly higher (HR: 12.44; CI: 4.62–33.49; HR: 6.01; CI: 1.70–21.27) compared to the reference group (0.2%). Conversely, the incidence in the medium/high-to-low-risk group was 0.5%, which showed no significant difference (HR: 1.91; CI: 0.45–8.14) compared to the reference group. Overall, the concordance index (C-index) was 0.911.

## 4. Discussion

This study demonstrated that follow-up ECGs prior to discharge, analyzed by an AI-enhanced model, provided superior predictive accuracy for 90-day all-cause mortality compared to the initial ECG obtained at ED arrival. Importantly, patients who were initially classified as low-risk but transitioned to medium- or high-risk through follow-up ECGs had significantly increased mortality, highlighting the dynamic nature of clinical risk and the value of serial assessment.

The enhanced predictive accuracy of the follow-up ECG conducted prior to discharge can be attributed to several key factors. Firstly, patients with unstable conditions presenting at the ED may have masked signs due to rising catecholamine levels in acute stress, which makes the initial ECG less reliable for risk prediction [14]. Secondly, a patient’s condition may fluctuate or deteriorate progressively during their stay, and the initial ECG may not capture these dynamic changes [15]. Thirdly, patients may become more stable after appropriate management, which the follow-up ECG can capture more effectively than the initial ECG. For instance, follow-up ECGs are often obtained after specific interventions in the ED, such as treatments for paroxysmal supraventricular tachycardia or atrial fibrillation with rapid ventricular response. These interventions stabilize the patient, allowing the follow-up ECG to more accurately reflect their true clinical condition at the time of discharge. The results of our study reveal that performing a follow-up ECG is more precise in risk stratification, ensuring that patients receive a more accurate assessment prior to discharge.

Our findings suggest that patients classified as low-risk at discharge can generally be safely sent home, whereas those in the medium/high-risk categories should be monitored closely, particularly those who were initially classified as low-risk. Bounce-back admissions and deaths within 7 days after ED discharge have been reported in approximately 2.6% and 0.05% of cases, respectively [16,17,18]. Clinical risk stratification tools, such as the HEART (History, ECG, Age, Risk Factors, and Troponin) score and the EDACS (Emergency Department Assessment of Chest Pain Score) are widely used to assess patients presenting with chest pain and suspected of acute coronary syndrome (ACS). However, both HEART and EDACS scores rely on discrete clinical and biochemical variables, which may not capture the dynamic physiological changes that occur over time [19,20]. Dynamic assessment for risk evaluation can better identify critical changes that impact clinical outcomes for patients in the ED. For example, the biomarkers N-terminal pro-brain natriuretic peptide and cardiac troponin require serial evaluations to track risk changes accurately, necessitating prompt treatment [21,22,23]. While initial ECGs provide valuable information, they may underestimate risk due to acute changes in a patient’s condition. Performing serial ECGs during the ED stay allows for more precise and timely risk assessment, offering a more accurate prediction of patient outcomes [24]. This approach reduces the risk of underestimation and improves patient outcomes by ensuring that individuals who require additional monitoring or intervention receive appropriate care.

In our study, of the 88 patients who died within the 90-day follow-up period, 27 (30.7%) deaths were attributed to pulmonary causes, 26 (29.5%) to malignancies, 13 (14.8%) to cardiogenic causes, 9 (10.2%) to infectious etiologies, and 13 (14.8%) to other causes. Notably, 9.1% of patients transitioned from the low-risk to the medium/high-risk group between their initial and follow-up ECGs. Two patients who presented to the ED in out-of-hospital cardiac arrest due to fatal hyperkalemia and severe gastrointestinal bleeding. Of the deaths, forty-one were potentially preventable and were associated with conditions such as ischemic heart disease, congestive heart failure, arrhythmias, chronic obstructive pulmonary disease, and hyperkalemia. The average time to death following ED discharge was 49.1 ± 21.6 days, with the shortest time being 12 days, indicating that these deaths were likely influenced by both acute and subacute underlying issues. These findings highlight the important role of AI-enabled ECGs in predicting short-term mortality risks of various etiologies, providing clinical support for timely referrals and follow-up treatments. Therefore, the implementation of follow-up AI-enabled ECGs in the ED is recommended, as it has the potential to enhance decision-making processes and optimize resource allocation.

Previous research has demonstrated the potential of AI-driven ECG models in accurately predicting adverse outcomes, such as mortality and major cardiac events including acute myocardial infarction, stroke, and heart failure [12,25]. ECGs are valuable for risk stratification, and their integration with other diagnostic tools, such as chest X-rays, can significantly enhance risk prediction in emergency settings [26,27]. In cases of acute myocardial infarction, the prognostic importance of the initial ECG is well established [28]. However, studies have shown that dynamic ECG changes offer superior predictive value for sudden cardiac death compared to baseline and static ECGs [29]. Additionally, dynamic ECGs have been shown to predict the risk of heart failure hospitalization [30]. AI-enabled ECGs in the ED significantly enhance risk stratification, enabling timely interventions and ensuring that high-risk patients receive appropriate care, which may ultimately reduce readmissions and improve patient outcomes [31,32]. Our study further underscores the importance of follow-up ECGs in the ED, as they capture dynamic changes in a patient’s condition, providing a more accurate risk stratification. This sequential approach emphasizes the benefits of repeated ECG assessments in predicting adverse outcomes and guiding clinical decision-making more effectively.

AI-enhanced ECG analysis has advanced significantly, with deep learning-based models and large language models (LLMs) demonstrating effectiveness in detecting cardiac conditions, predicting adverse events, and improving clinical decision-making [33,34]. However, the clinical adoption of these models is often limited by the need for extensive computational resources and structured multimodal datasets. Our AI-enabled ECG model relies exclusively on ECG waveform analysis, eliminating the need for additional clinical inputs while maintaining strong predictive performance. This approach offers key advantages, including real-time applicability and dynamic risk stratification, making it a potentially valuable tool for ED decision support. By providing timely and accurate risk assessment, our model has the potential to optimize ED workflows, improve patient disposition, and enhance clinical outcomes.

This study has several limitations that should be acknowledged. Firstly, the data were derived from a single medical center, which may limit the generalizability of our findings to other settings with different patient populations. Secondly, the study included patients who underwent two ECGs during their ED visit. These patients generally presented with cardiopulmonary symptoms, making them a higher-risk group that may not fully represent the broader ED population. This selection bias could have influenced the study’s outcomes and may limit the applicability of the findings to lower-risk patients. Thirdly, the retrospective design inherently carries the risk of biases related to data collection, missing information, and the inability to control for all confounding variables. Although we attempted to control the known confounders, there may be unmeasured variables that influenced the results. In particular, because mortality was captured only within our hospital records, some out-of-system deaths may have been missed and introduced bias. Additionally, the study did not evaluate the cost-effectiveness or feasibility of implementing AI-enabled ECG technology on a large scale in routine ED practice. Finally, while we included an analysis of ECG features and patient characteristics to assess model interpretability, future work should evaluate their clinical utility in prospective, multi-center settings.

## 5. Conclusions

The AI-enabled follow-up ECG demonstrated superior predictive accuracy compared to the initial ECG, highlighting the potential value of performing a follow-up ECG to enhance risk stratification for ED patients. The follow-up ECG, conducted prior to discharge, may provide a more accurate assessment of short-term mortality risk, particularly for those initially deemed low risk. Patients being considered for discharge from the ED could benefit from an additional ECG to support a more precise evaluation of their risk profile. Although AI-enabled ECG interpretation alone may not replace comprehensive risk assessment, it offers a practical tool to support clinical judgment, particularly in the dynamic ED environment, by supporting safer discharge decisions. Future research should aim to validate these findings across multiple centers, using prospective study designs and assessing the cost-effectiveness and real-world implementation of AI-enabled ECGs in diverse clinical settings.

## Figures and Tables

**Table 1:** Baseline demographic and clinical characteristics.

Variables	Total (*n* = 11,508)
Age, mean ± SD	63.64 ± 16.63
Gender, *n* (%)	
Male	6172 (53.6%)
Female	5336 (46.4%)
BMI, mean ± SD	24.86 ± 4.09
Triage level, *n* (%)	
1	324 (2.8%)
2	4359 (37.9%)
3	6441 (56.0%)
4	383 (3.3%)
5	1 (0.0%)
SBP, mean ± SD	142.30 ± 27.19
DBP, mean ± SD	79.78 ± 16.70
PULSE, mean ± SD	85.00 ± 24.28
SpO_2_, mean ± SD	98.28 ± 14.89
1st ECG mortality risk score, mean ± SD	68.84 ± 24.06
2nd ECG mortality risk score, mean ± SD	65.73 ± 24.15
Admission to discharge(hr.), mean ± SD	5.47 ± 4.39
Event [all-cause mortality within 90 days], *n* (%)	
Alive	10,248 (99.1%)
Death	88 (0.9%)

SD = standard deviation; BMI = body mass index; SBP = systolic blood pressure; DBP = diastolic blood pressure; SpO_2_ = peripheral capillary oxygen saturation.

**Table 2:** Characteristics of 3 categories of risk group predicted by AI-enabled ECG upon admission to the ED.

Variables	Low Risk	Medium Risk	High Risk	*p*-Value
Number, *n* (%)	8210 (71.3%)	2655 (23.1%)	643 (5.6%)	<0.001
Age, mean ± SD	60.59 ± 16.13	71.56 ± 15.09	69.98 ± 16.40	<0.001
Gender, *n* (%)				<0.001
Male	4535 (55.2%)	1302 (49.0%)	335 (52.1%)	
Female	3675 (44.8%)	1353 (51.0%)	308 (47.9%)	
BMI, mean ± SD	25.05 ± 4.04	24.40 ± 4.09	24.07 ± 4.52	<0.001
Triage level, *n* (%)				<0.001
1	126 (1.5%)	132 (5.0%)	66 (10.3%)	
2	2828 (34.4%)	1176 (44.3%)	355 (55.2%)	
3	4957 (60.4%)	1275 (48.0%)	209 (32.5%)	
4	298 (3.6%)	72 (2.7%)	13 (2.0%)	
5	1 (0.0%)	0 (0.0%)	0 (0.0%)	
SBP, mean ± SD	143.23 ± 26.38	141.67 ± 28.90	132.54 ± 28.53	<0.001
DBP, mean ± SD	80.74 ± 15.97	77.14 ± 17.95	77.97 ± 19.18	<0.001
PULSE, mean ± SD	81.50 ± 20.23	90.33 ± 27.67	108.21 ± 37.20	<0.001
SpO_2_, mean ± SD	98.37 ± 9.92	98.18 ± 25.60	97.60 ± 3.23	0.429
1st ECG mortality risk score, mean ± SD	59.26 ± 22.04	91.39 ± 2.80	98.08 ± 1.03	<0.001
2nd ECG mortality risk score, mean ± SD	58.53 ± 23.35	82.75 ± 14.73	87.36 ± 15.93	<0.001
Admission to discharge (hr.), mean ± SD	5.30 ± 4.30	5.79 ± 4.44	6.44 ± 5.16	<0.001
Events [all-cause mortality within 90 days], *n* (%)				<0.001
Alive	7292 (99.7%)	2395 (98.3%)	561 (96.1%)	
Death	24 (0.3%)	41 (1.7%)	23 (3.9%)	

SD = standard deviation; BMI = body mass index; SBP = systolic blood pressure; DBP = diastolic blood pressure; SpO_2_ = peripheral capillary oxygen saturation.

## Data Availability

The data presented in this study are available on request from the corresponding author due to ethical and privacy restrictions.

## Supplementary Materials

The following supporting information can be downloaded at: https://www.mdpi.com/article/10.3390/diagnostics15151874/s1, Figure S1: Visual interpretation of AI-enabled ECG mortality risk prediction.

## Abbreviations

The following abbreviations are used in this manuscript:

ECG	Electrocardiogram
EDs	Emergency departments
AI	Artificial intelligence
AUC	Area Under the Curve
KM	Kaplan–Meier
ROC	Receiver operating characteristic
HEART	History, ECG, Age, Risk Factors, and Troponin
EDACS	Emergency Department Assessment of Chest Pain Score
ACS	Acute coronary syndrome
LLMs	Large language models

## Appendix A

**Table A1:** Characteristics of patients classified as low-risk by AI-enabled ECG at ED admission.

Variables	Low–Low	Low–Medium	Low–High	*p*-Value
Number, *n* (%)	7527 (91.6%)	650 (8%)	33 (0.4%)	<0.001
Age, mean ± SD	59.73 ± 15.96	70.49 ± 14.69	61.15 ± 18.86	<0.001
Gender, *n* (%)				<0.001
Male	4228 (56.2%)	290 (44.6%)	17 (51.5%)	
Female	3299 (43.8%)	360 (55.4%)	16 (48.5%)	
BMI, mean ± SD	25.12 ± 4.02	24.30 ± 4.23	24.18 ± 4.33	<0.001
Triage level, *n* (%)				<0.001
1	105 (1.4%)	21 (3.2%)	0 (0.0%)	
2	2555 (33.9%)	264 (40.6%)	9 (27.3%)	
3	4592 (61.0%)	343 (52.8%)	22 (66.7%)	
4	274 (3.6%)	22 (3.4%)	2 (6.1%)	
5	1 (0.0%)	0 (0.0%)	0 (0.0%)	
SBP, mean ± SD	143.23 ± 26.19	143.49 ± 28.29	135.94 ± 30.38	0.296
DBP, mean ± SD	81.02 ± 15.91	77.80 ± 16.38	75.29 ± 16.65	<0.001
PULSE, mean ± SD	81.11 ± 19.44	85.46 ± 26.52	94.30 ± 35.42	<0.001
SPO_2_, mean ± SD	98.41 ± 10.30	97.82 ± 3.76	98.36 ± 2.18	0.337
1st ECG mortality risk score, mean ± SD	57.81 ± 22.07	75.68 ± 13.24	67.97 ± 20.81	<0.001
2nd ECG mortality risk score, mean ± SD	55.63 ± 22.20	90.08 ± 2.58	98.03 ± 1.07	<0.001
Admission to discharge (hr.), mean ± SD	5.23 ± 4.23	6.02 ± 5.01	5.77 ± 3.85	<0.001
Events [all-cause mortality within 90 days], *n* (%)				<0.001
Alive	6673 (99.8%)	593 (99.0%)	26 (92.9%)	
Death	16 (0.2%)	6 (1.0%)	2 (7.1%)	

**Table A2:** Characteristics of patients classified as medium-risk by AI-enabled ECG at ED Admission.

Variables	Medium–Low	Medium–Medium	Medium–High	*p*-Value
Number, *n* (%)	1223 (46.1%)	1310 (49.3%)	122 (4.6%)	<0.001
Age, mean ± SD	67.14 ± 15.89	75.43 ± 13.20	74.20 ± 13.91	<0.001
Gender, *n* (%)				0.003
Male	560 (45.8%)	686 (52.4%)	56 (45.9%)	
Female	663 (54.2%)	624 (47.6%)	66 (54.1%)	
BMI, mean ± SD	24.53 ± 4.13	24.29 ± 4.01	24.26 ± 4.56	0.369
Triage level, *n* (%)				0.109
1	52 (4.3%)	68 (5.2%)	12 (9.8%)	
2	525 (42.9%)	598 (45.6%)	53 (43.4%)	
3	612 (50.0%)	609 (46.5%)	54 (44.3%)	
4	34 (2.8%)	35 (2.7%)	3 (2.5%)	
5	0 (0.0%)	0 (0.0%)	0 (0.0%)	
SBP, mean ± SD	142.53 ± 28.53	141.23 ± 29.05	137.85 ± 30.74	0.190
DBP, mean ± SD	79.25 ± 17.85	75.35 ± 17.81	75.19 ± 18.33	<0.001
PULSE, mean ± SD	94.56 ± 30.66	86.87 ± 24.39	85.13 ± 22.96	<0.001
SPO_2_, mean ± SD	97.89 ± 3.18	98.48 ± 36.34	97.81 ± 2.81	0.839
1st ECG mortality risk score, mean ± SD	90.81 ± 2.79	91.79 ± 2.68	93.05 ± 2.78	<0.001
2nd ECG mortality risk score, mean ± SD	72.20 ± 15.92	91.20 ± 2.58	97.78 ± 0.90	<0.001
Admission to discharge (hr.), mean ± SD	5.54 ± 4.11	5.94 ± 4.62	6.61 ± 5.27	0.009
Events [all-cause mortality within 90 days], *n* (%)				<0.001
Alive	1102 (99.5%)	1183 (97.6%)	110 (94.8%)	
Death	6 (0.5%)	29 (2.4%)	6 (5.2%)	

**Table A3:** Characteristics of patients classified as high-risk by AI-enabled ECG at ED admission.

Variables	High–Low	High–Medium	High–High	*p*-Value
Number, *n* (%)	187 (29.1%)	269 (41.8%)	187 (29.1%)	<0.001
Age, mean ± SD	62.45 ± 16.87	72.24 ± 15.46	74.26 ± 14.70	<0.001
Gender, *n* (%)				0.229
Male	88 (47.1%)	143 (53.2%)	104 (55.6%)	
Female	99 (52.9%)	126 (46.8%)	83 (44.4%)	
BMI, mean ± SD	24.56 ± 4.10	23.85 ± 4.81	23.87 ± 4.49	0.252
Triage level, *n* (%)				0.604
1	21 (11.2%)	27 (10.0%)	18 (9.6%)	
2	105 (56.1%)	149 (55.4%)	101 (54.0%)	
3	57 (30.5%)	85 (31.6%)	67 (35.8%)	
4	4 (2.1%)	8 (3.0%)	1 (0.5%)	
5	0 (0.0%)	0 (0.0%)	0 (0.0%)	
SBP, mean ± SD	135.03 ± 27.89	134.07 ± 29.09	127.95 ± 27.97	0.040
DBP, mean ± SD	82.14 ± 18.93	77.45 ± 17.74	74.63 ± 20.66	0.001
PULSE, mean ± SD	123.79 ± 42.57	105.66 ± 33.65	96.10 ± 30.47	<0.001
SPO_2_, mean ± SD	98.27 ± 2.21	97.21 ± 3.94	97.48 ± 2.85	0.002
1st ECG mortality risk score, mean ± SD	98.14 ± 1.07	97.93 ± 1.00	98.24 ± 1.02	0.005
2nd ECG mortality risk score, mean ± SD	68.69 ± 18.77	92.74 ± 2.72	98.27 ± 1.03	<0.001
Admission to discharge (hr.), mean ± SD	6.02 ± 4.53	6.61 ± 5.78	6.60 ± 4.81	0.427
Events [all-cause mortality within 90 days], *n* (%)				<0.001
Alive	175 (100.0%)	241 (96.8%)	145 (90.6%)	
Death	0 (0.0%)	8 (3.2%)	15 (9.4%)	
