# Supplementary material for: Prognostic Significance of AI-Enhanced ECG for Emergency Department Patients
Source: Diagnostics (Basel). 2025 Jul 25;15(15):1874. doi: 10.3390/diagnostics15151874 (PMC12346629; doi:10.3390/diagnostics15151874)
Supplement: Supplementary file 1 [file diagnostics-15-01874-s001.zip › diagnostics-3707589-supplementary.pdf]

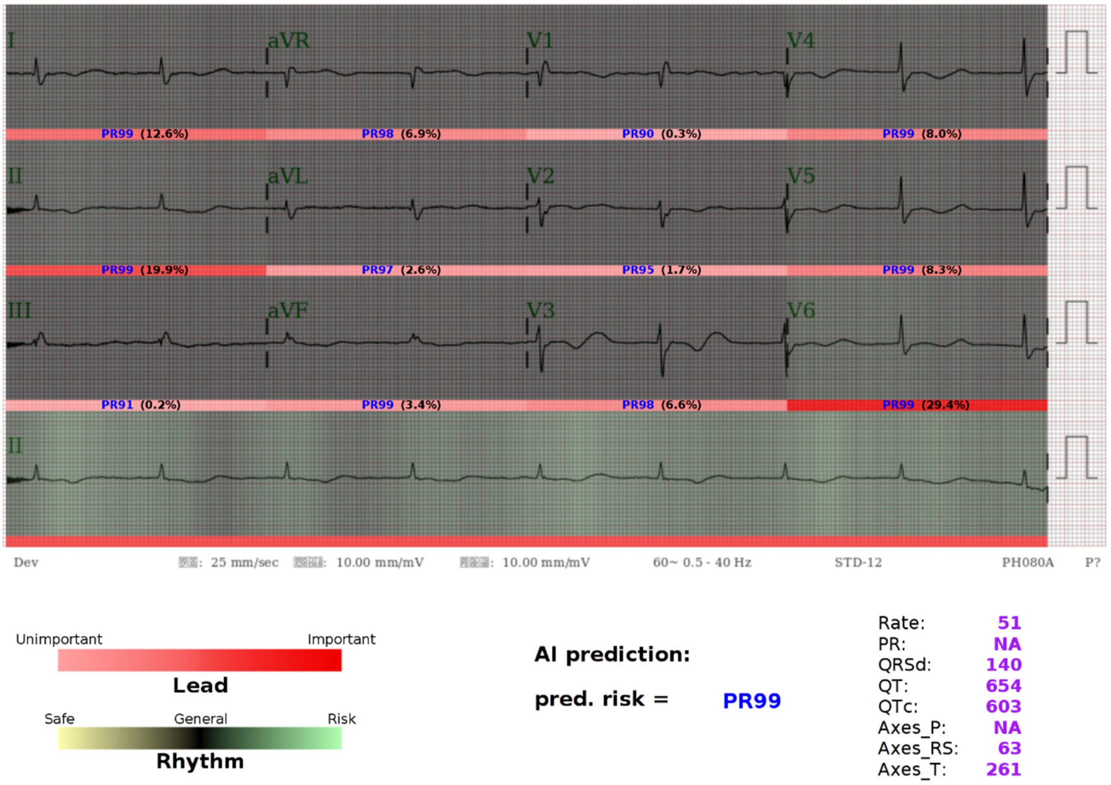

Figure S1. Visual interpretation of AI-enabled ECG mortality risk prediction.

This illustration depicts the AI-derived 90-day mortality risk from a 12-lead ECG. Each lead shows a percentile risk score (e.g., PR99), with darker red shading indicating higher model contribution. Rhythm strips are color-coded from yellow (low risk) to green (high risk) to reflect rhythm-related risk assessments. Standard ECG metrics are listed on the right. The final prediction indicates PR99, suggesting extremely high risk. This figure serves as a visual aid to enhance clinician understanding of AI model outputs.
